# Sustainable Ultralightweight U-Net-Based Architecture for Myocardium Segmentation

**DOI:** 10.3390/jcm14227971

**Published:** 2025-11-10

**Authors:** Jakub Filarecki, Dorota Mockiewicz, Agata Giełczyk, Tamara Kuźba-Kryszak, Roman Makarewicz, Marek Lewandowski, Zbigniew Serafin

**Affiliations:** 1 Faculty of Telecommunications, Computer Science and Electrical Engineering, Bydgoszcz University of Science and Technology, 85-796 Bydgoszcz, Poland; 2 Department of Radiotherapy, Centre of Oncology in Bydgoszcz, 85-796 Bydgoszcz, Poland; 3 Department of Oncology and Brachytherapy, Ludwik Rydygier Collegium Medicum in Bydgoszcz, Nicolaus Copernicus University in Torun, 85-067 Bydgoszcz, Poland

**Keywords:** computer vision, Green AI, MRI images, myocardium, segmentation, U-Net

## Abstract

**Background:** Medical image segmentation is essential for accurate diagnosis and treatment planning. The U-Net architecture is widely regarded as the gold standard, yet its large size and high computational demand pose significant challenges for practical deployment. **Methods:** Real data (MRI images) from hospital patients were used in this study. We proposed a novel lightweight architecture tailored specifically for myocardium (cardiac muscle) segmentation. **Results:** We presented results comparable to state-of-the-art methods in terms of IoU and Dice coefficients. Nonetheless, the results achieved are much more favorable from the perspective of AI’s sustainable development. The proposed architecture ensured the following average results: IOU = 0.7889 and Dice = 0.8780 using 263 k parameters and a total of 6.24 G FLOPs. **Conclusions:** The proposed schema can potentially be used to support radiologists in improving the diagnostic process. The presented approach is efficient and fast. Most promisingly, the reduction in the model’s complexity is significant compared to the state-of-the-art methods.

## 1. Introduction

According to the World Health Organization (WHO), the leading global causes of death in 2021 were primarily cardiovascular diseases (such as stroke or ischemic heart disease) and respiratory conditions (including COVID-19 and chronic obstructive pulmonary disease) (see https://www.who.int/news-room/fact-sheets/detail/the-top-10-causes-of-death, accessed on 1 October 2025). Additionally, the WHO estimates a projected shortage of 11 million health workers by 2030, especially in low- and lower-middle-income countries (https://www.who.int/health-topics/health-workforce, accessed on 1 October 2025). These two factors help to explain why computer scientists, particularly data scientists, have become increasingly involved in developing machine learning (ML)-based diagnostic tools. Such tools can assist medical professionals in analyzing images like X-rays, positron emission tomography (PET), computed tomography (CT), magnetic resonance imaging (MRI), and ultrasound (US), potentially leading to a reduction in cardiovascular disease incidence and alleviating the burden on overextended healthcare systems in various countries.

In this paper, we focus on a fundamental step in any deep learning-supported diagnostic tool: image segmentation. Numerous studies have demonstrated that proper image segmentation is essential for achieving accurate, precise, and fair results. As mentioned in [1], accurately identifying the location and size of a tumor is crucial for selecting the optimal treatment plan as it assists doctors in determining the surgical margins and minimizing damage to healthy tissue. Moreover, as emphasized in [2], human-based segmentation can involve a significant degree of subjectivity. Manual segmentations performed by the same operator on the same images at different times may vary. Therefore, automatic segmentation is preferable—not only in terms of time and effort but also regarding accuracy, repeatability, and reproducibility.

The heart is a vital organ that is essential for maintaining the body’s physiological balance, known as homeostasis [3]. Its contractile function is carried out by the myocardium (i.e., cardiac muscle, or myo)—tissue composed of specialized cells called cardiomyocytes. These cells exhibit unique structural and physiological characteristics that enable the heart to generate sufficient force to ensure proper blood perfusion to tissues and organs throughout the body. In a healthy individual, the left ventricle (LV) plays a key role by pumping oxygen-rich blood to the rest of the body. The walls of the LV are typically thick and composed of dense cardiac muscle fibers to support this function. The condition of the heart muscle can be assessed using imaging techniques such as magnetic resonance imaging (MRI). Cardiac MRI provides detailed information about the anatomy of the heart chambers, valves, the size of the heart, blood flow through major vessels, and surrounding structures. It is a valuable tool for detecting or monitoring heart conditions, including heart failure, myocardial infarction (heart attack), and other cardiovascular diseases.

This study investigates the segmentation of the myocardium in MRI scans. As noted in [4], segmenting the myocardium can be more challenging than segmenting the left and right ventricles. The main contributions of this article are as follows:

1. We propose a novel ultralightweight U-Net-based model tailored for myocardium segmentation;
2. We introduce a new dataset with manually segmented cardiac muscle areas, validated by specialists, which is publicly available from GitHub;
3. We demonstrate comparable segmentation accuracy in terms of IoU and Dice coefficients, alongside significant reductions in model complexity and parameter count;
4. Our work aligns with the Green AI trend by considering not only accuracy but also model size, computational complexity, and operational efficiency. We provide a thorough quantitative analysis of the model’s sustainability using FLOPs and parameter counts.

## 2. Related Work

### 2.1. Medical Image Segmentation

Different types of images can be used in medical ML-based diagnosis, including X-rays, PET, CT, MRI, and US. As mentioned in [5], segmentation provides critical details that are necessary for further analysis. Previously, traditional image processing techniques were used, such as edge enhancement and detection. Currently, ML-based techniques have become more widely adopted.

The review article in [6] enumerates possible approaches to deep learning-based medical image segmentation: convolutional neural network (CNN) [7], fully convolutional network (FCN) [8], recurrent neural network (RNN) [9], and autoencoder (AE) [10]. CNNs are widely used not only for segmentation but also for the proper classification of the segmented parts of an image.

Recently, the Segment Anything Model (SAM) [11] has gained significant attention as a powerful and versatile vision segmentation model. It can generate diverse and detailed segmentation masks based on user prompts. Despite its strong performance regarding natural images, recent studies also show [12,13] that it can underperform on medical image segmentation.

In [14], the authors present an automatic and accurate coarse-to-fine segmentation framework for myocardial pathology, integrating U-Net++ and EfficientSeg architectures. Initially, U-Net++ with deep supervision is employed to perform coarse segmentation by delineating cardiac structures from multi-sequence cardiac magnetic resonance (CMR) images. The resulting segmentation maps, combined with the original three-sequence CMR data, are subsequently refined using the EfficientSeg-B1 model to identify pathological regions such as myocardial scar and edema.

Ref. [15] presents an innovative approach to cardiac segmentation in short-axis MRI images. The proposed method comprises three main stages: (1) extraction of a region of interest, (2) segmentation of the myocardium (myo) and left ventricular cavity (LVC) using the EAIS-Net architecture, and (3) segmentation of the right ventricle (RV) utilizing the IRAX-Net architecture. Notably, the accuracy of myocardium segmentation is the most challenging.

The authors in [16] introduce an innovative nnFormer architecture designed for 3D medical image segmentation. The model combines convolutional operations with self-attention in an interleaved manner, incorporating both local and global volumetric self-attention to effectively capture long-range spatial dependencies. In experiments, nnFormer significantly outperforms previous transformer-based models and reduces computational complexity compared to nnUNet.

Ref. [17] describes a myo segmentation framework for sequences of cardiac MRI scanning images. The proposed method combines conventional neural networks and recurrent neural networks to incorporate temporal information between sequences to ensure temporal consistency. The evaluation of the framework is performed using the ACDC dataset.

The authors in [18] introduce a novel adaptation of the MLP (Multi-Layer Perceptron)-Mixer architecture for cardiac MRI segmentation. The authors enhance the MLP-Mixer layers to improve global feature extraction, thereby increasing segmentation accuracy. A shifted-window-partition layer is incorporated to capture local interactions and enrich feature representation. The experimental results show that the proposed Swin-MLP model outperforms state-of-the-art methods in cardiac MRI segmentation, especially in myocardium segmentation (reported Dice over 92%).

In [19], the authors present an AI model capable of detecting normal regions of the left ventricular cavity, normal myocardium, and other normal tissues. The proposed workflow employs CLAHE (Contrast Limited Adaptive Histogram Equalization) for preprocessing, effectively enhancing local contrast and preserving fine image details. The reported accuracy of myocardium segmentation is 91.28%.

Recent developments have led to the introduction of the Mamba architecture. It has demonstrated promising capabilities across various computer vision tasks and has been adapted in numerous implementations, including MF-Mamba [20], Swin-UMamba [21], and SegMamba [22]. Its ability to balance long-range dependency modeling with computational efficiency makes it particularly well-suited for medical image segmentation tasks, where both precision and scalability are crucial.

The state-of-the-art research demonstrates that myocardium segmentation is a crucial step in computer-aided classification of heart diseases. However, most methods proposed in the literature rely on large and resource-intensive architectures. Our motivation was to develop a lighter and more eco-friendly model.

### 2.2. Green AI

With the rapid growth of AI applications across all areas of life, the ecological impact can no longer be ignored. As mentioned in [23], the term Green AI refers to AI-based systems that are capable of maximizing energy efficiency and reducing their environmental impact. Various metrics can be used to assess a model’s environmental impact, including carbon emissions, electricity usage, and elapsed real time. However, these parameters can vary significantly depending on hardware, geographic location, or time of measurement [24].

Alternatively, the total number of model parameters can be evaluated. Nevertheless, different algorithms utilize parameters in different ways—such as by increasing model depth versus width—meaning that models with a similar number of parameters may still perform vastly different amounts of computation. To address this, the FLOP metric (floating-point operation) has been introduced, estimating the total number of floating-point operations required during model training. This parameter is hardware-agnostic and is based on the number of additions and multiplications performed.

In [25], reduction in model size is identified, among other approaches, as a potential technique for enhancing the sustainability of AI-based systems. On the other hand, the authors in [26] highlight precision/energy trade-off monitoring and hyperparameter tuning as commonly employed strategies.

## 3. Materials and Methods

### 3.1. Dataset

The dataset consists of cardiac MRI images from 22 patients. In total, the dataset includes 269 images that have been preprocessed and standardized to a resolution of 224×224 pixels. Each image in the dataset has been carefully and manually annotated to highlight relevant cardiac structure. These annotations were subsequently reviewed and validated by a medical expert to ensure clinical accuracy and reliability. Originally stored in DICOM (.dcm) format, the images have been converted to PNG (.png) files for the purposes of simplifying integration into common deep learning workflows and improving accessibility [27]. The train–test split was performed based on the number of patients rather than the number of images. MRI scans from a single patient were assigned entirely to one group—either training or testing—in order to prevent data leakage and overfitting. Each patient’s folder contained several images from both before and after treatment. According to our own analysis and consultations with an oncologist, there were no visible differences between these scans, so it should not affect the results or the quality of the segmentation model. These two types of scans were taken at different time points, which helped to increase the diversity of the dataset. For each patient, the data is organized into the following structure: Therapy Stage: A—Images taken before the therapeutic intervention. B—Images taken after therapy to assess changes. Image Type: MAG (Magnitude)—Standard anatomical images showing tissue structure. PS (Phase)—Phase-contrast images encoding blood flow velocity.

**Figure 1:**
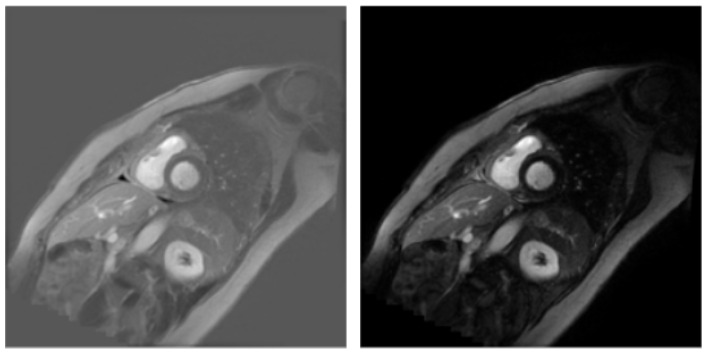
PS and MAG images from the dataset.

MAG and PS images represent the same anatomical regions but are processed differently; therefore, to increase the diversity of the dataset for both image types, separate masks were manually created for each rather than reusing the same mask. Examples of corresponding PS and MAG images from the dataset are presented in Figure 1.

All experiments were conducted on the same dataset, enhanced by random horizontal flips (50% probability) and random rotations (up to 30°). In our work, data augmentation was not intended to increase the effective size of the training set per se as simple replication through augmentation did not improve performance. Instead, augmentation was applied primarily to increase the diversity of the training samples. This diversity helps to prevent rapid overfitting and improves the model’s ability to generalize rather than simply enlarging the dataset.

All MRI images were acquired with the approval of the Ethics Committee of Nicolaus Copernicus University in Toruń and Ludwik Rydygier Collegium Medicum in Bydgoszcz (decision no. KB132/2019, approved on 29 January 2019). The authors confirm that all experiments were conducted in accordance with relevant guidelines and regulations. Informed consent was obtained from all participants and/or their legal guardians.

### 3.2. Architecture

**Figure 2:**
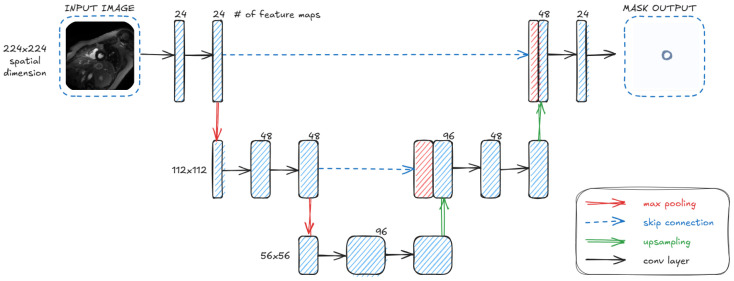
The pipeline of the proposed architecture. The model consists of an encoder and a decoder connected by skip connections. Each part contains double convolution layers, followed by max pooling or upsampling.

Our architecture not only consists of fewer layers than the original U-Net architecture presented in [28] but also has a significantly reduced number of parameters. The new lightweight model consists of two downsampling and two upsampling blocks with double convolutional layers between the input and output layers, as presented in Figure 2.

The input in this architecture was grayscale images with a size of 224 × 224 pixels. As in every U-Net-based architecture, the model begins with the encoder part. The input tensor is processed by a double convolution block consisting of two convolutional layers with a kernel size of 3 and padding of 1, each followed by batch normalization and an ReLU activation function. In the output, we obtain a 24-channel tensor. Next, our architecture consists of two downsampling blocks, each of them including a double convolution layer followed by maxpooling, which reduces the size by 2. At the bottom of the U-shaped architecture, our tensor size is expanded to 96 channels, size 56×56 .

In the decoder part, there are two upsampling layers applied, each of which consists of an upscaling part, adding the output of the parallel layer in the encoder section, and applying a double convolution layer in the end. Finally, the outer layer operations are executed. The tensor is processed by the convolution layer with a kernel size set to 1, which at the end produces output with one channel for the binary segmentation, size 224×224. Summing up, the model consists of two down and two up layers, among the in and out layers, which allows for reduction in the number of parameters to 263,257.

As the final step in setting up the model, we performed hyperparameter tuning. Because our architecture is lightweight, the training process was relatively fast. This allowed us to test multiple variants of hyperparameters, such as batch size (2 up to 128), learning rate ( $1\times 10^{5}$ up to $1\times 10^{-1}$ ), optimizer type (Adam, SGD, and others), etc., in order to identify the best-performing configuration.

## 4. Results

### 4.1. Evaluation Metrics

The proposed method was compared with state-of-the-art methods using two classic metrics, namely by means of IoU (Intersection over Union) and Dice coefficient. The coefficients are expressed via Equation (1) and Equation (2), respectively, where A—ground truth mask, B—predicted mask. Dice coefficient places greater emphasis on correctly predicted positives, which is particularly useful in medical imaging contexts where the region of interest (e.g., the myocardium) is typically very small compared to the entire image. On the other hand, IoU tends to penalize small errors more severely than Dice.

(1) $$IoU=\frac{\left|A\cap B\right|}{\left|A\cup B\right|}$$

(2) $$Dice=\frac{2\;\cdot |A\cap B|}{\left|A|+|B\right|}$$

Moreover, both the FLOPs and the total number of parameters were evaluated. FLOPs (floating-point operations) represent the total number of arithmetic operations required to perform a computational task. They provide a deterministic estimate of the computational workload by assigning defined costs to two basic operations: addition and multiplication. The FLOP count for a given model can typically be estimated before training. The total number of parameters, on the other hand, refers to the complete set of weights and bias terms that must be learned during training.

### 4.2. Obtained Results

The training loop used the Adam optimizer and a hybrid loss function composed of binary cross-entropy (BCE) and Dice loss, weighted equally. The dataset was split into a 90:10 train–test ratio and loaded using a batch size of 32. To ensure result stability and mitigate the influence of random initialization, we conducted 10 independent training runs on different data splits, each initialized with a unique random seed. The reported metrics represent the averaged performance across these runs. The whole training process took a maximum of 100 epochs, usually less since we used early stopping to prevent overfitting. During the experiments, we used the cloud-based architecture provided by Google. As such, specific hardware characteristics (e.g., CPU/GPU model and memory architecture) are not easily identified or fixed. We ensured that the same architecture was used in each performed experiment. We compared the results of several models on the provided test data:

1. ResNet18-U-Net: This model follows the standard U-Net decoder architecture but uses a ResNet18 encoder. It contains approximately 14.3 million parameters. By using a ResNet18 backbone, the model can benefit from transfer learning, leveraging features learned from large-scale datasets such as ImageNet. The decoder is a custom upsampling path designed to match the feature maps from the ResNet layers via skip connections.
2. Original U-Net: A widely used baseline architecture with four downsampling and four upsampling blocks, each composed of double convolution layers [28]. It was originally designed for biomedical image segmentation.
3. Small U-Net: A simplified version of U-Net architecture with only two downsampling and two upsampling blocks.
4. UwU-Net (Proposed): A lightweight version of U-Net with a reduced number of layers and parameters, making it almost twice as light as Small U-Net.

Since a proprietary dataset was used in this study, it was not possible to directly compare our model with existing approaches known from the literature. Therefore, we re-implemented selected state-of-the-art models and trained them on our dataset to ensure a fair comparison.

Our starting point was the classic ResNet18-U-Net model as it is readily available in the PyTorch library. We tried using pretrained weights, but the model performed better when trained from scratch on our dataset (79.0% IoU and 88.0% Dice). This behavior can be explained by the substantial domain gap between natural images and the medical data used in this study, which reduced the effectiveness of transfer learning. Although the dataset consisted of 269 samples, extensive data augmentation was applied to increase its diversity and improve generalization. Testing on an external dataset was not performed as the available datasets differed significantly in acquisition protocols and image characteristics, making direct comparison unreliable. Unfortunately, the ResNet18-U-Net model was quite heavy, with more than 14 million parameters. Next, we implemented the original U-Net model, which turned out to handle the segmentation problem almost exactly the same (79.0% IoU and 87.9% Dice) despite its simpler architecture.

After that, we decided to reduce the number of layers to only two downsampling and two upsampling blocks between the input and output layers. This resulted in the Small U-Net model. The results were quite promising, although the metrics dropped slightly (77.7% IoU and 87.0% Dice).

Within the series of experiments, we decided to reduce the number of parameters, which made our version of U-Net even lighter, with only about 250k parameters. This resulted in a slight improvement in metrics (78.9% IoU and 87.8% Dice), making it as good as the basic U-Net and the complex ResNet18-U-Net while maintaining a light and easy-to-train architecture. Other reductions, both in the number of layers and parameters, resulted in a significant drop in metrics.

Some experiments with the larger U-Net versions resulted in an improvement in metric scores when switching from the ReLU activation function to LeakyReLU. However, in our ultralight model, this change actually led to slightly worse results. That is why the UwU-Net architecture with ReLU is the preferred solution. Although slightly larger, it achieves better results and maintains the eco-friendly design goal. The difference in effectiveness is best illustrated by the lowest evaluation scores: IoU 77% vs. 71% and Dice 86% vs. 82%, respectively (presented in Table 1 and Table 2).

The results of the experiments are presented in Table 1 and Table 2, including values for IoU and Dice. Table 3 presents the FLOPs and total number of parameters. In addition to reporting the mean values (from 10 runs of experiments) of the Dice coefficient and IoU, we also present descriptive statistics, including the minimum, maximum, and standard deviation for each evaluated model. These measures provide insight into both the central tendency and variability in model performance, allowing for a clearer comparison of stability and robustness across architectures. We conducted a statistical analysis, and the resulting *p*-values exceeded 0.05, indicating that there are no statistically significant differences between our method and the state-of-the-art solutions. This demonstrates that our results in terms of Dice and IoU are comparable, while the model complexity has been reduced significantly.

**Figure 3:**
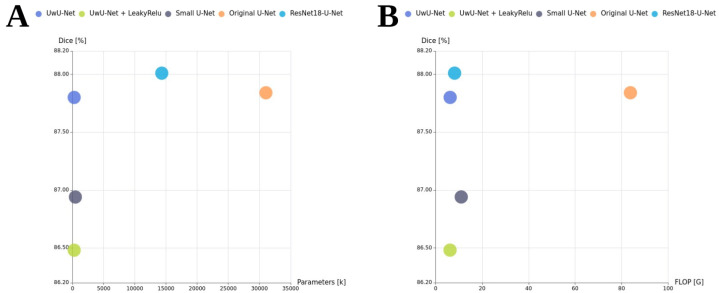
Model green evaluation: (**A**) Dice vs. total number of parameters; (**B**) Dice vs. FLOPs.

The Green AI evaluation was performed by comparing the trade-off between the values: Dice vs. FLOPs and Dice vs. total number of parameters. This comparison is presented in Figure 3. The Figure 3 provides a clear perspective on the trade-off between segmentation accuracy and computational complexity. In both parts of the Figure 3A,B, the most promising model in terms of technological sustainability is the one located closest to the top-left corner, representing the model that ensures the highest performance (Dice score) while maintaining the lowest complexity (FLOPs and number of parameters). In both cases, our proposed UwU-Net was the most environmentally friendly model. Specifically, in Figure 3A, UwU-Net achieves one of the highest Dice scores while requiring a fraction of the parameters compared to the original U-Net, demonstrating exceptional parameter efficiency. Similarly, Figure 3B shows that UwU-Net attains high accuracy with markedly lower FLOP counts than the baseline, aligning well with the principles of Green AI. Notably, ResNet18-U-Net, while more complex in terms of FLOPs and parameter count, delivers slightly lower Dice performance, and the original U-Net, despite having the largest computational footprint (>80 G FLOPs and >30 M parameters), only marginally outperforms lighter models in Dice, suggesting diminishing returns with increased complexity.

From a clinical standpoint, small differences in Dice or IoU (e.g., 0.2–0.5 percentage points) may not translate into a meaningful diagnostic impact, especially considering that the proposed model produces smoother and more anatomically consistent boundaries compared to some manual annotations. The reduced size and complexity of UwU-Net make it particularly suitable for real-time or resource-constrained settings, such as on-device inference in MRI scanners or deployment in clinics with limited computational infrastructure. These characteristics underline the model’s practical applicability and potential to facilitate efficient and accurate myocardial segmentation in diverse clinical environments.

## 5. Discussion

**Figure 4:**
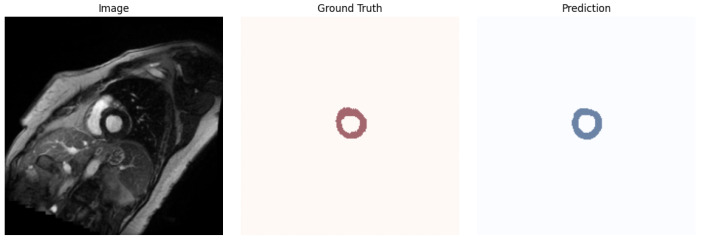
Example of segmentation results (from left to right): MRI image, ground truth mask, and model prediction. Although both masks are generally similar, they differ in fine details, particularly along the mask boundaries. These differences arise mainly due to the irregular edges present in the manual annotations provided by the labelers, whereas the model prediction tends to produce smoother contours.

The use of AI in medicine presents significant opportunities, but it also introduces the imperative to ensure transparency, explainability, and understandable reasoning in decision-making systems [29]. Figure 4 shows, from left to right, an exemplary image from the dataset, the corresponding ground truth, and the model’s prediction. The mask predicted by the model clearly reveals internal irregularities corresponding to the papillary muscles. Some experts, as well as specialized software, may also classify these structures as part of the myocardium. Notably, manual masks are annotated at the pixel level and often exhibit irregular edges due to inherent annotation variability. In contrast, our model tends to produce masks with smoother, more regular, and anatomically plausible contours. This difference highlights the model’s ability to generalize beyond the pixel-level noise present in human annotations.

An important factor affecting myocardium segmentation accuracy is the presence of papillary muscles within the ventricular cavity. In our dataset, these structures were annotated inconsistently due to differing practices among expert radiologists: some included papillary muscles within the myocardium mask, while others excluded them. This heterogeneity introduces inherent variability in the ground truth annotations, which in turn impacts the evaluation of model performance. Our results demonstrate that the model can capture such internal irregularities, but the variability in annotation protocols highlights the need for standardized guidelines in future studies. Addressing the segmentation of papillary muscles explicitly may be a valuable direction for further improving model robustness and clinical applicability.

**Figure 5:**
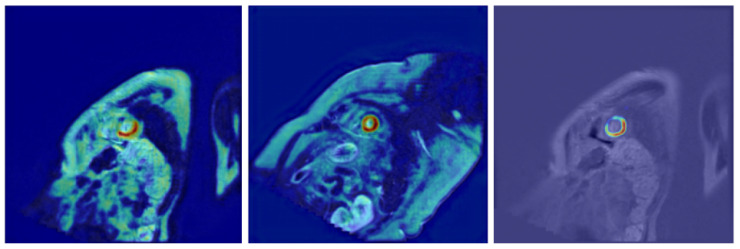
Examples of less promising results. Grad-CAM visualizations show that the model generally identifies the myocardium region accurately; however, in some cases, the predicted regions are narrower than expected or deviate from the typical oval shape.

In Figure 5, some examples of less promising results are presented. The Grad-CAM visualizations indicate that the model generally localizes the myocardium region accurately, with high correspondence to the ground truth annotations. However, in certain cases, the predicted regions appear narrower than expected or deviate from the typical oval morphology of the myocardium. These discrepancies are likely related to subtle variations in image contrast or anatomical presentation, and, while they may slightly affect quantitative metrics, the overall segmentation quality remains clinically acceptable. It is worth noting that even specialists performing manual segmentations encountered occasional difficulties in accurately delineating the myocardium when the image quality was lower, which may also explain some of the observed differences.

Table 4 presents the Dice and IoU scores (where possible) obtained for our proposed architecture alongside selected state-of-the-art (SOTA) methods from the literature. The experiments were conducted on a specific dataset whose characteristics differ notably from commonly used benchmarks. Therefore, direct comparison with SOTA approaches should be interpreted with caution as differences in data distribution and annotation protocols may affect the reported metrics. Nevertheless, our architecture demonstrates significant potential, particularly in terms of computational efficiency and alignment with the emerging trend of Green AI. Unfortunately, the complexity of the SOTA architectures remains unknown as information on FLOPs and the number of parameters is not available.

## 6. Conclusions

In this article, we present a novel ultralightweight U-Net-based architecture for myocardium segmentation on MRI images. The reported results (in terms of IoU and Dice coefficients) are comparable to the state of the art. However, we were able to minimize the number of trained parameters without losing prediction quality. The proposed architecture can be treated as a green example of AI since we implemented various techniques used for sustainable AI model development, namely hyperparameter tuning, early stopping of training, precision/energy trade-off monitoring, and model size reduction.

Based on the conducted research, several promising directions for future development have been identified:

1. Further model explainability—In the current work, we have integrated explainability techniques into our architecture, incorporating Grad-CAM visualizations to highlight the image regions that most significantly influenced the segmentation output. These visualizations not only improve trust in the model’s decisions but also provide valuable feedback for clinicians by revealing patterns that are consistent with anatomical structures. Future work may extend this approach by exploring additional methods, such as integrated gradients or layer-wise relevance propagation, to provide complementary perspectives on model reasoning and further enhance clinical interpretability.
2. Further energy-aware optimization—Although the proposed model demonstrates competitive performance, further optimization with respect to energy efficiency remains an important issue for future work. Advanced hyperparameter tuning or model pruning strategies could be employed to reduce the computational cost (e.g., FLOPs and total number of parameters) while potentially maintaining or even improving the segmentation performance. This is particularly relevant in the context of sustainable AI and deployment in resource-constrained environments.
3. Extension to diagnostic classification systems—The current segmentation architecture could be extended to support classification tasks. For instance, by analyzing the segmented myocardium, the system could assist in detecting specific cardiac pathologies (e.g., myocardial infarction, fibrosis, or inflammation) based on extracted textural or morphological features. Integrating segmentation with classification may provide a comprehensive diagnostic pipeline that enhances clinical decision-making.

## Figures and Tables

**Table 1:** Mean (Avg.), minimum (Min.), maximum (Max.), and standard deviation (Std.dev.) of the IoU coefficient for the proposed and state-of-the-art models.

Model	Avg.	Min.	Max.	Std.dev.
UwU-Net (proposed)	0.7889	0.7686	0.8173	0.0168
UwU-Net + LeakyRelu (proposed)	0.7697	0.7075	0.8139	0.0286
Small U-Net	0.7769	0.7347	0.8097	0.0195
Original U-Net	0.7896	0.7534	0.8154	0.0176
ResNet18-U-Net	0.7909	0.7787	0.8007	0.0076

**Table 2:** Mean (Avg.), minimum (Min.), maximum (Max.), and standard deviation (Std.dev.) of the Dice coefficient for the proposed and state-of-the-art models.

Model	Avg.	Min.	Max.	Std.dev.
UwU-Net (proposed)	0.8780	0.8618	0.8938	0.0109
UwU-Net + LeakyRelu (proposed)	0.8648	0.8203	0.8920	0.0198
Small U-Net	0.8694	0.8343	0.8888	0.0153
Original U-Net	0.8784	0.8466	0.8926	0.0142
ResNet18-U-Net	0.8801	0.8713	0.8879	0.0058

**Table 3:** FLOPs and total number of parameters for the proposed and state-of-the-art models.

Model	FLOP [G]	Parameters [M]
UwU-Net (proposed)	6.24	0.263
UwU-Net + LeakyRelu (proposed)	6.22	0.263
Small U-Net	11.02	0.467
Original U-Net	83.79	31.042
ResNet18-U-Net	8.16	14.321

**Table 4:** SOTA comparison.

Ref.	Year	Architecture	Dataset	Result
[18]	2024	Swin-MLP	ACDC	Dice = 0.9290
[15]	2024	EAIS-Net	ACDC, M&Ms	Dice = 0.8454, IoU = 0.7578
[16]	2023	nnFormer	ACDC	Dice = 0.8958
[17]	2022	CNN + RNN	ACDC	Dice up to 0.7656
proposed	2025	UwU-Net	own dataset	Dice = 0.8780, IoU = 0.7889

## Data Availability

The dataset is available from GitHub: https://github.com/PBS-Bydgoszcz/SMR.git, accessed on 1 October 2025.
